# Exome sequencing expands the mutational spectrum of SPG8 in a family with spasticity responsive to l-DOPA treatment

**DOI:** 10.1007/s00415-013-7044-6

**Published:** 2013-07-24

**Authors:** Conceição Bettencourt, Huw R. Morris, Andrew B. Singleton, John Hardy, Henry Houlden

**Affiliations:** 1 Department of Molecular Neuroscience, UCL Institute of Neurology, Queen Square, London, WC1N 3BG UK; 2 Laboratory of Neurogenetics, National Institute on Aging, National Institutes of Health, Bethesda, USA

Dear Sirs,

Hereditary spastic paraplegias (HSPs) are clinically and genetically heterogeneous. Pure forms are characterized primarily by progressive spasticity and weakness of the lower limbs. Complicated forms, however, involve neuronal systems other than corticospinal tracts, namely peripheral nerves, and sensory or cerebellar pathways [2]. At least 52 loci and 31 causative genes are known, and thus a gene-by-gene diagnostic approach is becoming impractical. For the 19 autosomal dominant forms (AD-HSPs), 11 genes are known, with SPG4 being by far the most common subtype (40–45 % AD-HSP cases) [4]. The vast heterogeneity in HSP makes a genetic diagnosis difficult and expensive. Many research groups have used gene panels or targeted sequencing but the rapid growth and frequent identification of new genes makes this difficult.

**Fig. 1:**
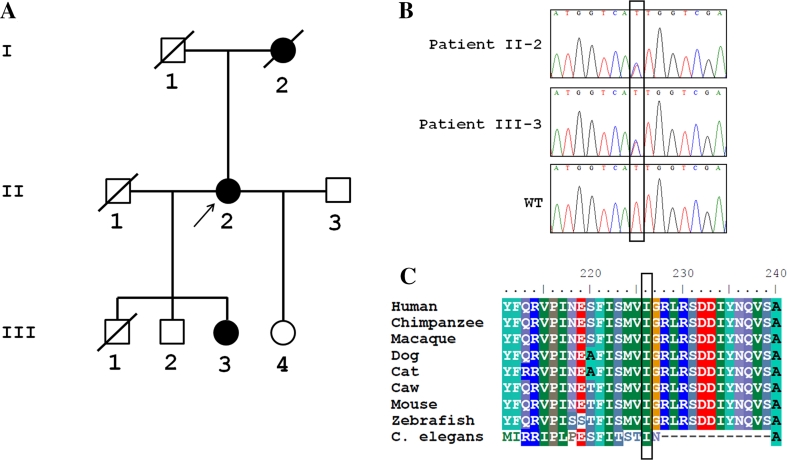
**a** Pedigree of a UK family with spastic paraplegia. The *arrow* points to the proband. *Black* and *white symbols* indicate affected and unaffected individuals, respectively. **b** Electropherograms illustrating the novel *KIAA0196* missense variant (c.677T > C, p.I226T) identified in our family. **c** Sequence alignment of WASH complex subunit strumpellin, which is encoded by the *KIAA0196* gene, showing the conservation between species in the mutated position

We studied a female patient presenting familial spastic paraplegia with sensory axonal neuropathy, compatible with AD inheritance (Fig. 1a). At the age of 10 years she developed problems walking and subsequently had many surgical procedures on her feet. After a pregnancy at the age of 42, she became more tired and walking slowed down with more pain. She has significant spasticity and sensory symptoms in her feet, burning, redness, occasionally whiteness, and she is often in extreme pain and discomfort. Both brain and spinal cord MRI scans were normal. She was started on l-DOPA (Co-Careldopa 25/100 mg tds), as one of the differential diagnoses was dopamine responsive dystonia, which was very helpful for her spasms and sensory symptoms in her feet. Additionally, she had bladder urgency and skin features to suggest autonomic dysfunction, and her gait was typical of spastic paraplegia. Her daughter reported early problems presenting at the age of 19, with pain in her feet and in the back of her legs, spasticity, cramps and sensory symptoms. Her gait showed a slight inversion of the right foot on walking. Interestingly, the daughter had tried l-DOPA in the form of Co-Careldopa 25/100 mg with benefit to her spasticity similar to her mother. The proband’s mother was also thought to have feet problems, with cold and stiff painful feet as well as abnormal sensation but her presentation was late in her mid-50s with very slow deterioration.

Although whole-exome sequencing is still expensive, and not everywhere available, it has been shown to be a useful tool for the diagnosis of complicated HSP [1]. Using this approach, we identified a novel *KIAA0196* missense variant in the proband (c.677T>C, p.I226T). By Sanger sequencing, her daughter was shown to have the same variant (Fig. 1b). This variant is predicted as damaging by SIFT, is located in a conserved residue (Fig. 1c), and is not present in more than 200 in-house exomes or in public databases, such as dbSNP, 1000 Genomes Project, and the Exome Variant server (http://evs.gs.washington.edu/EVS/).

The *KIAA0196* gene (MIM#610657) is responsible for SPG8 (MIM#603563), a rare AD-HSP, and encodes for strumpellin, a protein that participates in the WASH complex, acting at the interface between actin regulation and endosomal membrane dynamics [5]. There are only two reports of mutations in this gene [3, 8], one of them recently published in this journal. Overall, there are seven SPG8 families, usually with pure phenotypes, and four pathogenic mutations identified worldwide (Table 1). Our pedigree is compatible with an AD-HSP, and the variant we found in the *KIAA0196* gene, segregating with the phenotype, is most likely causing HSP. The response to l-DOPA is interesting and may suggest an overlap of the functional pathway of *KIAA0196* and dopamine. Although the knowledge of the underlying mutation does not change the therapeutic strategy, it is valuable on what concerns genetic counseling. We therefore identified the eighth SPG8 family and the fifth mutation by making use of next-generation sequencing. Furthermore, we report a complicated phenotype, with an earlier onset than those previously reported (Table 1), also expanding the clinical spectrum of SPG8.

**Table 1:** Mutations in the *KIAA0196* gene identified in HSP families

Mutation	Phenotype							Number of families	Reference
	Onset (years)	Pure/complicated	Lower limbs				Other		
			Spasticity	Hyperreflexia	Weakness	Babinski sign			
c.677T>C, p.I226T	10–55	Complicated	+	+	Distal	+	Sensory axonal neuropathy, dopamine responsive spasticity, urinary urgency	1	Present study
c.1491A>G, p.N471D	22–29	n.a.	n.a.	n.a.	n.a.	n.a.	n.a.	1	[8]
c.1937G>C, p.L619F	18–26	Pure	+	+	+	+	Bladder dysfunction	1	[7, 8]
c.1956G>T, p.V626F	20s–60s	Pure	n.a.	+	n.a.	n.a.	Impaired vibratory sense, urinary urgency	4	[6, 8]
c.2087G>C, p.G696A	21–57	Pure	+	+	+	+	Impaired vibratory sense, urinary urgency	1	[3]

+ present, *n.a.* information not specified in the reference
